# Mesoporous-Structure MOF-14-Based QCM *p*-Xylene Gas Sensor

**DOI:** 10.3390/nano13111743

**Published:** 2023-05-26

**Authors:** Zhiheng Ma, Tongwei Yuan, Yu Fan, Yang Chen, Yueling Bai, Jiaqiang Xu

**Affiliations:** 1 NEST Lab, Department of Physics, Department of Chemistry, College of Science, Shanghai University, Shanghai 200444, China; mzh139863@163.com (Z.M.); twkiller@outlook.com (T.Y.); fanyu666@shu.edu.cn (Y.F.); 2 Key Laboratory of Organic Compound Pollution Control Engineering (MOE), School of Environmental and Chemical Engineering, Shanghai University, Shanghai 200444, China; shucy@shu.edu.cn

**Keywords:** quartz crystal microbalance, adsorption enthalpy, metal−organic frameworks (MOFs), VOC detection

## Abstract

In this work, a facile synthesis method was adopted to synthesize MOF-14 with mesoporous structure. The physical properties of the samples were characterized by PXRD, FESEM, TEM and FT-IR spectrometry. By coating the mesoporous-structure MOF-14 on the surface of a quartz crystal microbalance (QCM), the fabricated gravimetric sensor exhibits high sensitivity to *p*-toluene vapor even at trace levels. Additionally, the limit of detection (LOD) of the sensor obtained experimentally is lower than 100 ppb, and the theoretical detection limit is 57 ppb. Furthermore, good gas selectivity and fast response (15 s) and recovery (20 s) abilities are also illustrated along with high sensitivity. These sensing data indicate the excellent performance of the fabricated mesoporous-structure MOF-14-based *p*-xylene QCM sensor. On the basis of temperature-varying experiments, an adsorption enthalpy of −59.88 kJ/mol was obtained, implying the existence of moderate and reversible chemisorption between MOF-14 and *p*-xylene molecules. This is the crucial factor that endows MOF-14 with exceptional *p*-xylene-sensing abilities. This work has proved that MOF materials such as MOF-14 are promising in gravimetric-type gas-sensing applications and worthy of future study.

## 1. Introduction

As one of important volatile organic compounds (VOCs), xylene is widely used in various chemical production industries and indoor decoration [1,2]. Meanwhile, as a member of BTEX (benzene, toluene, ethylbenzene, xylene), xylene has high carcinogenicity [3,4,5], causing irritation to skin and mucous membranes and inducing an anesthetic effect on the central nervous system. Inhalation of high-concentration *p*-xylene in a short time can cause serious irritation to the eyes and upper respiratory tract, as well as congestion of the conjunctiva and pharynx; excessive inhalation can cause agitation, convulsion, coma, etc. Neurasthenia syndrome, hepatomegaly, dry skin, chapped skin, dermatitis and other symptoms can occur after long-term exposure [6]. On 27 October 2017, in the list of carcinogens published by the International Agency for Research on Cancer of the World Health Organization, xylene was classified as Category Ⅲ, indicating a possible cancer risk [7]. Therefore, the realization of real-time detection and leakage prevention of xylene is not only the basis of ensuring industrial production safety, but also guarantees employees’ health.

There are pre-existing detection methods for detecting benzene-series molecules, such as gas chromatography–mass spectrometry [8,9], fluorescence analysis [10] and so on. However, these detection methods not only depend on expensive equipment, but also require professionals to carry out a series of complex and time-consuming operations to complete the detection procedure. At the same time, their use is limited due to the disadvantages associated with cumbersome test methods, large volume of test equipment, which is not conducive to in situ rapid detection, and so on. As an important detection method, gas sensors, including semiconductor gas sensors [11], electrochemical gas sensors [12], catalytic combustion gas sensors [13], infrared gas sensors [14], solid electrolyte gas sensors [15] and so on, have been widely studied by scientists in virtue of many advantages such as fast response, convenient operation and low price [16,17]. 

Quartz crystal microbalance (QCM) gas sensors [18,19,20,21,22,23,24] are a new technology developed in recent years. Compared with traditional semiconductor sensors, QCM sensors feature high sensitivity, fast response time, room working temperature and easy portability. Hence, it has attracted much attention from many researchers. QCM gas sensors mainly utilize quartz crystal microbalances as the working platform, converting the mass change of quartz crystal surfaces into frequency change according to the Sauerbrey equation [25]; the frequency change is then collated into digital signal by a computer. Specialized QCM gas sensors for specific gases can be manufactured by preparing different sensitive coatings.

QCM sensors usually use polymers, metal oxides and mesoporous and microporous materials as sensitive materials. As a category of microporous materials, metal–organic frameworks (MOFs) have attracted the attention of gas sensor researchers because of their ultra-high specific surface area, large porosity and adjustable pore structures. There have been many reports on the application of MOF materials in gas sensors, including in the detection of CO by Ni-MOF-74 [26], in the testing of xylene by HKUST-1 [27], in the sensing of VOC gases by HKUST-1 [28] and pyridine by MIL-101 (Al) [29] and so on. Through the analysis of previous literature reports [30,31], we found that samples with different MOF pore structure may have different gas-sensing performances.

As a classical microporous material, MOF-14 [32,33,34] has a stable structure and a mild metal site Cu^2+^. Therefore, it could possess strong adsorption on the electron-rich Lewis alkaline gases such as those of the benzene series. As examples of Lewis alkaline gases with conjugated electrons, members of the benzene series can not only have acid–base interactions with the Cu^2+^ of MOF-14, but also have a π–π stacking effect with the benzene ring in the ligand, which enables MOF-14 to be used as a potential benzene-series-sensitive material [35]. In previous studies [36], we have demonstrated the excellent benzene-series sensitivity of MOF-14 and used it as a sensitive material to prepare a gas sensor for benzene vapor. However, the detection limit of the sensor still needs to be further improved, because it can only detect 400 ppb of benzene vapor, which is still high for the toxic and harmful benzene series. Therefore, we tried to improve the sensitivity of MOF-14 to the benzene series by changing its pore structure. Summarizing previous experience, we designed and synthesized MOF-14 with different pore sizes, and studied the effect of the pore structure of the sensitive material on the detection of the benzene series. The experimental results show that the pore size of MOF-14 grows along with an increase in the pH of solution. When the pore structure of MOF-14 changes from microporous to mesoporous, the response value of MOF-14 to the benzene series greatly improves and shows excellent selectivity to *p*-xylene. Then, We further characterized the mesoporous MOF-14 material and evaluated in a series of *p*-xylene-sensing experiments. The sensor exhibited high sensitivity (340 Hz @ 1 ppm *p*-xylene), good repeatability, long-term stability and satisfactory selectivity to *p*-xylene gas.

The research in this paper proves that increasing the pore size of MOF materials can significantly improve their sensitivity performance. In the process of changing the pore size of porous materials, the increase in pore size weakens the steric hindrance between different gas molecules, thereby shifting the selectivity of sensitive materials towards gas molecules from molecular size control to adsorption heat effect control. This discovery can not only be applied to MOF materials, but also has guiding significance for other microporous and mesoporous materials such as mesoporous carbon, mesoporous silicon, COF materials, etc. In addition, this discovery is also of great significance for the identification of aromatic hydrocarbon vapor molecules in urban air and the chemicals industry.

## 2. Materials and Methods

### 2.1. Materials

Copper (II) nitrate heMi (pentahydrate) (Cu(NO_3_)_2_·2.5H_2_O, 98.0%) and 1,3,5-Tri(4-carboxyphenyl) benzene (H_3_BTB, 98.0%) were obtained from Alfa Aesar. Benzene (99.0%), toluene (99.5%), xylene (99.0%) and N, N-dimethylformamide (DMF, 99.0%) were purchased from Shanghai Chemical Reagent Co., Ltd., Shanghai, China. Methanol and sodium acetate were obtained from Sinopharm Co., Ltd., Beijing, China.

### 2.2. Synthesis of MOF-14 with Microporous Structure

The synthesis of microporous MOF-14 is based on previous literature [35], and the synthesis details can be found in the Supporting Materials.

### 2.3. Synthesis of MOF-14 with Mesoporous Structure

First, 11.0 mg H_3_BTB (0.025 mmol) and 11.6 mg Cu(NO_3_)_2_·2.5H_2_O (0.05 mmol) were dissolved into a 20 mL mixture of ethanol and DMF with volume ratio 1:1, and then 16.4 mg (0.2 mmol)) of sodium acetate was weighed and added into the mixed solution. Then, the mixture obtained was transferred into a Teflon-lined stainless-steel autoclave (25 mL) and heated in an electric oven at 353 K for 5 h. The precipitate was taken out and washed several times with DMF to remove any unattached products. Finally, the product was dried by Critical Point Dryers.

### 2.4. Fabrication of QCM Sensors and Measuring Equipment

The preparation of the QCM sensor refers to the previous work of our research group [37,38,39], replaced with MOF-14 samples. Testing of the QCM sensor included three steps: (1) baseline obtained by purging with high-purity synthetic air; the gas to be tested was introduced only after the baseline was stable (when the frequency drift does not exceed 5 Hz). (2) A certain concentration of gas calculated in advance was measured and extracted with a micro-sampler and quickly injected into the test chamber. When the adsorption capacity reached a maximum and the curve remained stable, the adsorption–desorption achieved an equilibrium state. At this time, the frequency change is the response value. (3) Finally, we purged with high-purity synthetic air to desorb the adsorbed gas from the sensing materials, and the frequency value returned to baseline. We repeated the above operations to test the gas at different concentrations to obtain continuous response curves. The testing chamber was set into a heating oven to keep the temperature stable and all measurements were conducted at room temperature (25.0 ± 1.0 °C). The relative humidity was controlled to about 40% using a dehumidifier.

### 2.5. Characterization

The instruments used for material characterization included SEM, TEM, XRD, F-IR, etc. Specific testing details can be found in the Supporting Materials.

## 3. Results

### 3.1. Characterization of MOF-14

**Figure 1:**
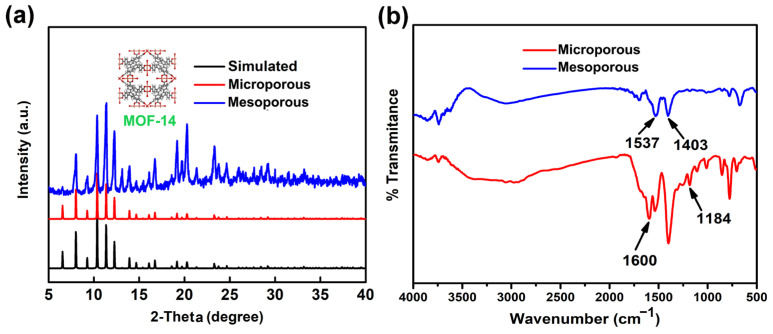
(**a**) XRD experimental and simulated patterns [40] of MOF-14; (**b**) FT-IR spectra of two kinds of MOF-14.

Figure 1 shows the XRD patterns and FT-IR spectra of the MOF-14 samples. By comparing XRD patterns of two kinds of MOF-14 samples, it can be seen that all strong peak positions basically coincide. Water and ethanol were used as solvents at room temperature in the synthesis of microporous MOF-14, which resulted in a slower coordination rate between the organic ligands and the central metal ions, resulting in a longer crystallization process to grow relatively complete crystals. In comparison, the mesoporous MOF-14 was synthesized by the hydrothermal method, enabling high temperature and pressure to accelerate the crystallization process of MOF-14. In addition, the presence of CH_3_COONa also promoted the deprotonation process of ligand H_3_BTB to increase the reaction speed. The factors listed above combined to produce defects in the MOF-14 crystal, leading to the emergence of mesopores. In both synthesis processes, coordination reactions only occurred between Cu and H_3_BTB; hence, no intermediate phases were generated. Therefore, both synthesized MOF-14 crystals have pure phases, the only difference being whether there are defects in the crystals. Further, the crystal structure of the mesoporous MOF-14 was partly destroyed, leading to a slightly wider XRD diffraction peak.

As shown in Figure 1b, the peaks of $\tilde{v}$ = 1537 cm^−1^ and $\tilde{v}$ = 1403 cm^−1^ belong to the *v*_as_(-COO^−^) and *v*_s_(-COO^−^) stretching vibrations of BTB^3−^, indicating the successful coordination of Cu^2+^. Additionally, the peaks of $\tilde{v}$ = 1600 cm^−1^ and $\tilde{v}$ = 1184 cm^−1^ could be attributed to the stretching vibrations of C=O and C–OH, which were not found from the spectrum of mesoporous MOF-14. This illustrated that the H_3_BTB of mesoporous MOF-14 finished deprotonation completely.

**Figure 2:**
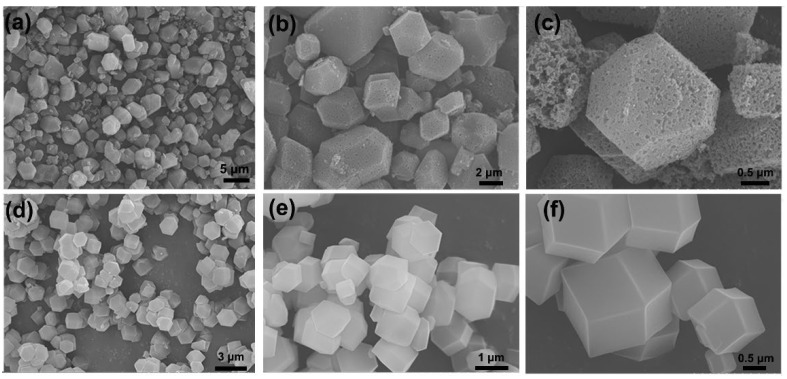
SEM images (**a**–**c**) of mesoporous MOF-14 and SEM images (**d**–**f**) of microporous MOF-14.

**Figure 3:**
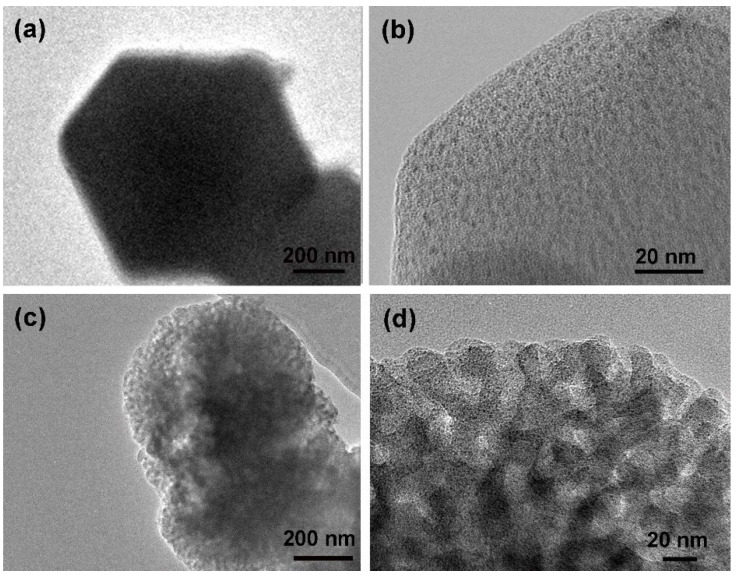
(**a**) Low-power TEM diagram and (**b**) High-power TEM diagram of microporous MOF-14; (**c**) Low-power TEM and (**d**) High-power TEM of mesoporous MOF-14.

The different morphologies of the MOF-14 are shown in Figure 2. Figure 2a–c are SEM images of mesoporous MOF-14 at different magnifications, and its mesoporous structure can be clearly observed under low magnification. Compared with the regular morphology of microporous MOF-14 (Figure 2d–f), the morphology of mesoporous MOF-14 is more non-uniform and the crystal size is larger. Combined with the TEM pictures in Figure 3, the different pore structures of the two kinds of MOF-14 are further characterized. In low-magnitude TEM images shown in Figure 3a, the micropores of the microporous MOF-14 cannot be observed, and are only seen at high magnification (Figure 3b). In contrast, the pore structure of mesoporous MOF-14 is obvious at low magnification.

**Figure 4:**
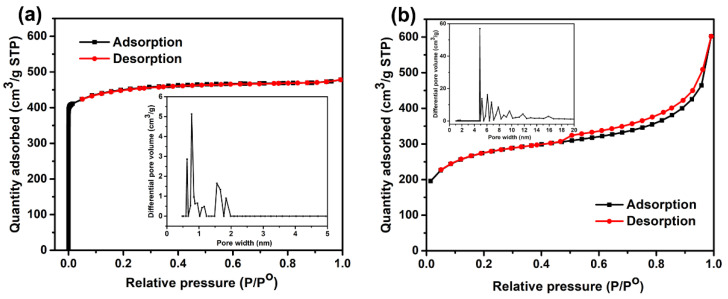
Adsorption and desorption isotherms of nitrogen (at 77 K) for MOF-14 microcrystals with different pore structures: (**a**) microporous MOF-14; (**b**) mesoporous MOF-14.

The porosity of the two kinds of MOF-14 was investigated by N_2_ adsorption–desorption measurements as shown in Figure 4. The microporous MOF-14 shows type I isotherms of IUPAC classification with a steep uptake at a low relative pressure (*P*/*P*_0_ < 0.05), demonstrating that the micropores of the microporous MOF-14 are primarily responsible for its high surface area as shown in Figure 4a. In contrast, the N_2_ sorption isotherms of mesoporous MOF-14 show discrete hysteresis loops, typical of cage-type pores (Figure 4b). The gradual uptake at a relative pressure of 0.4–0.95 is due to a capillary condensation of N_2_ gas in a porous structure consisting of mesopores with different sizes (the inset picture). Meanwhile, for better comparison, we created a table to list the BET-specific surfaces, Langmuir surfaces, pore sizes and pore volumes of the two MOF-14 samples. From the Table 1, it is observed that the specific surface area reduced and pore size increased obviously when the structure of MOF-14 transitioned from microporous to mesoporous. Specifically, the pore volume of mesoporous MOF-14 is double that of microporous MOF-14, which could provide better gas-sensing performance.

### 3.2. Sensing Performance of MOF-14-Based QCM Sensors

After loading MOF-14 onto a QCM sensor, we can calculate the mass of the material using the Sauerbrey equation, as well as the mass of the gas adsorbed by the sensitive material. Details of the Sauerbrey equation can be found in the Supporting Material.

**Figure 5:**
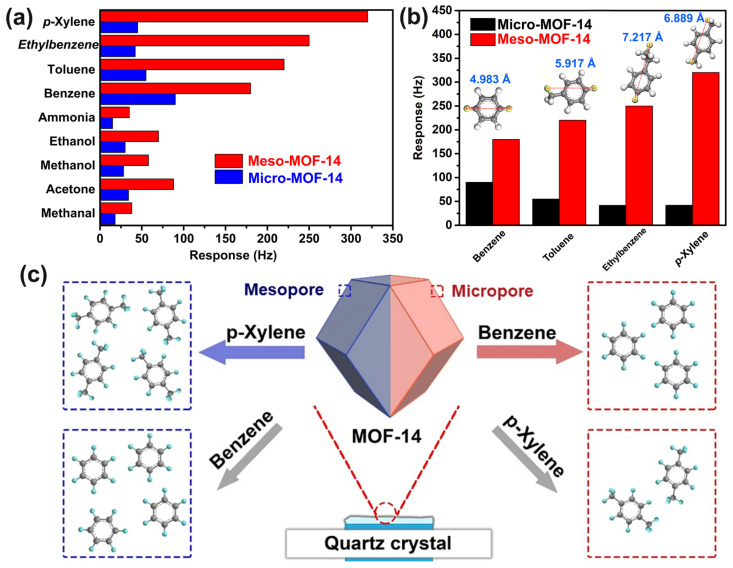
(**a**) Gas selectivity test of two kinds of MOF-14 materials; (**b**) The relationship between the diameter and the response of MOF-14 to different benzene homologues; (**c**) Schematic diagram of spatial steric effect of MOF-14 with different pore structures.

An important index of the sensor is its selectivity to the target gas. Therefore, the selectivity of two kinds of MOF-14-based QCM sensors were investigated, and are shown in Figure 5a. By comparing the response values of the QCM sensor to nine kinds of gases with 1 ppm concentration, it is obvious that the response of mesoporous MOF-14 to all test gases is greatly improved compared with that of microporous MOF-14, and the proportion of the increase in the response value to the benzene series is much higher than that of other interfering gases. In addition, the selectivity of MOF-14 changed significantly. The mesoporous MOF-14 has the highest response to *p*-xylene, while the microporous MOF-14 showed the highest response to benzene. For other non-benzene-series gases such as methanol, ethanol, acetone, formaldehyde and ammonia, the difference in response value is caused only by the relative molecular weight. Because the relative molecular weight difference of these small molecules is not great, they do not show special selectivity. Considering the similar competitive adsorption among members of the benzene series, the reason for the greater response of mesoporous MOF-14 to *p*-xylene than other members of the benzene series is that the existence of two methyl (electron-donating) groups results in a higher adsorption enthalpy than benzene, toluene and ethylbenzene.

To further explain the reason for the selective alteration of MOF-14, the molecular diameters of BTEX members and corresponding response values are shown in Figure 5b. It can be seen from the figure that the molecular diameter of benzene is 4.98 Å, toluene’s is 5.91 Å, xylene’s is 6.88 Å and ethylbenzene’s is 7.21 Å, and the response value of microporous MOF-14 to BTEX members decreases with increasing molecular diameter, while mesoporous MOF-14 increases with an increasing number of methyl groups on the benzene ring. It is well-demonstrated that the appearance of mesopores leads to the weakening of the steric hinderance effect of BTEX members in MOF-14, which greatly enhances the sensitivity performance of MOF-14.

In an extremely ideal case, microporous MOF-14 with a pore size of 1–2 nm can accommodate 2–4 benzene molecules or 1-2 *p*-xylene molecules, which can accommodate about 2 times as many benzene molecules as *p*-xylene, while mesoporous MOF-14 with a pore size of 5–10 nm can accommodate 10–20 benzene molecules and 7–14.5 *p*-xylene molecules. Considering the intermolecular repulsion force, the number of benzene and *p*-xylene molecules could be approximately equal; thus, the selectivity is determined by the adsorption enthalpy of BTEX members and MOF-14 (Figure 5c).

Therefore, we can determine that the pore size has an important impact on the gas-sensing performance of MOF-14 to BTEX members. Part of this specific adsorption comes from its unique structure and composition (containing a large number of metal cations and benzene rings), and the other part comes from the pore size effect (steric hindrance). Therefore, the MOF-14 sample with appropriate pore size can be used as a potential *p*-xylene-sensing material.

**Figure 6:**
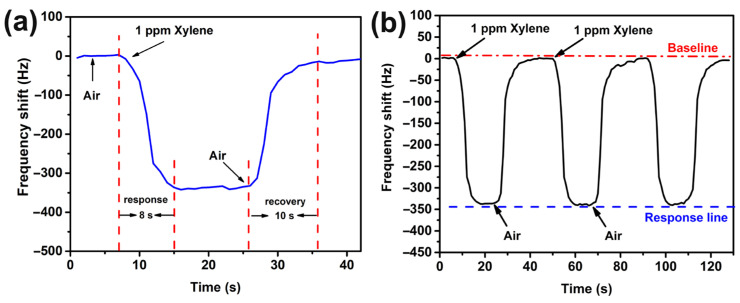
(**a**) Response–recovery curve; (**b**) Cycling stability of mesoporous MOF-14-based QCM to 1 ppm *p*-xylene vapor.

Figure 6a shows the response–recovery curve of a *p*-xylene gas sensor made from mesoporous MOF-14. It can be seen from the figure that when 1 ppm *p*-xylene is injected into the test chamber, the response value increases rapidly within 5 s and reaches a maximum value at 8 s, which is almost stable. After the air is re-introduced, the *p*-xylene is blown away by the air. With a decrease in gas concentration, it can recover to 90% of the baseline at 8 s, and return to the baseline position after 10 s. The curve shows that the sensor has fast response and recovery speeds. Figure 6b displays the cyclic stability test of the QCM sensor. For three consecutive tests involving 1 ppm *p*-xylene gas, the response values are 340 Hz, 343 Hz and 341 Hz, respectively. The difference between the three tests is less than 5 Hz, proving good cyclic stability of the sensor.

**Figure 7:**
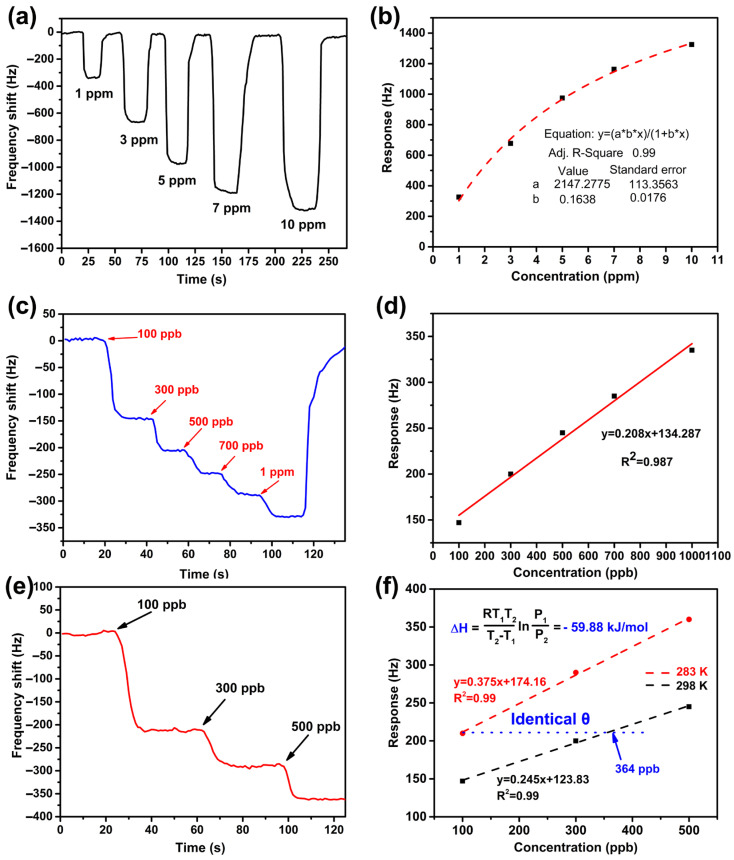
(**a**) Response and recovery curves of MOF-14-based QCM to *p*-xylene vapor from 1 to 10 ppm; (**b**) Corresponding Langmuir adsorption fitting; (**c**) Dynamic responses of MOF-14-based QCM to sub-1 ppm *p*-xylene vapor at 298 K; (**d**) Linear fitting of low-concentration *p*-xylene vapor; (**e**) Dynamic responses of MOF-14-based QCM to sub-1 ppm *p*-xylene vapor at 283 K; (**f**) In terms of the experimental results in (**c**,**e**), two isotherms are plotted to calculate the value of adsorption enthalpy (ΔH).

Figure 7a exhibits the test curve of the concentration gradient of the sensor. It can be seen from the figure that when 1 ppm *p*-xylene gas is injected, the response is about 340 Hz. When the *p*-xylene concentration is increased to 3 ppm, the response value is about 664 Hz, showing that the difference caused by the change in concentration of 2 ppm is 324 Hz. When the inlet concentration is 5 ppm *p*-xylene, the response value is 975 Hz, and the response caused by the 2 ppm concentration change is 311 Hz. The same additional 2 ppm *p*-xylene was introduced, but the increment in the response was relatively small compared with the first 2 ppm response value. This shows that the adsorption capacity is larger and the adsorption process is the faster at the beginning of contact with *p*-xylene gas. In the subsequent process, the increasing trend of the response value decreases with an increase in concentration, which is similar to the Langmuir adsorption model. Therefore, we fitted the adsorption capacity at different points with the Langmuir adsorption model to produce Figure 7b. The R^2^ value after fitting is 0.99, indicating that the adsorption process of mesoporous MOF-14 is Langmuir adsorption. Figure 7c,d shows the continuous response test and linear fitting under ultra-low concentrations at 298 K. The linear fitting equation is y = 0.208x + 134.287.15; R^2^ = 0.987. It shows that the response increases linearly with concentration level, and through LOD = 3σ/S equation, the logical limit of detection can be calculated (σ = 3.86, s = 0.208), 58 ppb. At the same time, this straight line is also used to calculate the adsorption enthalpy in the adsorption process by the variable-temperature weighing method.

The above experimental results show that the QCM sensor coated with mesoporous MOF-14 has exceptional sensing performances for low concentrations of *p*-xylene vapor. This is demonstrated by its excellent selectivity and sensitivity, good reversibility and repeatability, significant stability and ideal response–recovery time. These excellent sensing properties are attributed to the strong interaction between *p*-xylene and the MOF-14 material.

In order to prove the existence of a specific interaction between *p*-xylene and mesoporous MOF-14 material, a temperature-varying experiment was used to obtain the adsorption curve of the sensor at low temperatures by changing the working temperature. Combined with the illustration in Figure 7d, the adsorption enthalpy of mesoporous MOF-14 to *p*-xylene can be calculated through the Clausius–Clapeyron relation as described in a previous report [41,42,43]. It is well-known that the parameters of thermodynamics play a crucial role in evaluating the properties of sensing materials, especially the adsorption enthalpy (ΔH). According to traditional physical–chemical theory [44], when the absolute value of ΔH is higher than 80 kJ/mol, there is strong chemical adsorption between the sensing material and the measured gas molecules, which is irreversible. In comparison, when the value is lower than 40 kJ/mol, it indicates weakly selective reversible adsorption. Therefore, considering the selectivity and reversibility, the ΔH of an ideal sensing material should be in the range of 40 kJ/mol to 80 kJ/mol [45].

Figure 7e shows the adsorption curve of a mesoporous MOF-14-coated QCM sensor for *p*-xylene vapor detection in the concentration range of 100~500 ppb at 283 K. By fitting and combining the curve with Figure 7c, we obtain Figure 7f showing that the partial pressure of *p*-xylene vapor increases linearly with concentration. According to the Clausius–Clapeyron relation, ΔH is calculated to be −59.88 kJ/mol, meaning that there is a reversible chemisorption between mesoporous MOF-14 and *p*-xylene molecules. The results demonstrate that Cu-MOF-74 is a good candidate for *p*-xylene vapor detection by QCM sensor.

## 4. Conclusions

In summary, MOF-14 materials with different pore structures were synthesized by controlling the pH of solution. It is found that the pore size has an important influence on the *p*-xylene sensitivity of the fabricated MOF-14-based QCM sensors. The mechanism that affects the gas sensitivity is discussed in terms of acid–base interactions, specific surface area and pore size. The results show that mesoporous-structure MOF-14 shows the best sensing performance for *p*-xylene vapor detection. Its response value to 1 ppm *p*-xylene gas is as high as 340 Hz. At the same time, the detection limit of the sensor can reach 58 ppb, along with a short response–recovery time (8 and 10 s). Through the calculation of its adsorption enthalpy, it is also proven that there is an ideal interaction between mesoporous MOF-14 and *p*-xylene molecules. Additionally, the selectivity, repeatability and stability of the sensor meet the market requirements of gas sensors. Hence, using this material as a coating is promising in developing a fast, sensitive and portable QCM *p*-xylene sensor to ensure industrial production and human safety.

## Figures and Tables

**Table 1:** Summary of the tested physical parameters of the two MOF-14 materials.

Materials	S_BET_ (m^2^/g)	S_Langmuir_ (m^2^/g)	Pore Volume (cm^3^/g)	Pore Size (nm)
Meso-MOF-14	880	1063	0.93	4.23
Micro-MOF-14	1237	1548	0.54	1.75

## Data Availability

Not applicable.

## Supplementary Materials

The following supporting information can be downloaded at: https://www.mdpi.com/article/10.3390/nano13111743/s1.
