# Supplementary material for: Mesoporous-Structure MOF-14-Based QCM p-Xylene Gas Sensor
Source: Nanomaterials (Basel). 2023 May 26;13(11):1743. doi: 10.3390/nano13111743 (PMC10254762; doi:10.3390/nano13111743)
Supplement: Supplementary file 1 [file nanomaterials-13-01743-s001.zip › nanomaterials-2396479-supplementary.pdf]

Supplementary Materials

Mesoporous structure MOF-14-based QCM *p*-xylene gas sensor

Zhiheng Ma [1,†], Tongwei Yuan [1,†], Yu Fan [1], Yang Chen [2], Yueling Bai [1,*] and Jiaqiang Xu [1,*]

[1] NEST lab, Department of Physics, Department of Chemistry, College of Science, Shanghai University, Shanghai 200444, China.

[2] Key Laboratory of Organic Compound Pollution Control Engineering (MOE), School of Environmental and Chemical Engineering, Shanghai University, Shanghai 200444, China.

E-mail: MZH139863@163.com (Z.-H. Ma), twkiller@outlook.com (T.-W. Yuan), fanyu666@shu.edu.cn (Y. Fan), shucy@shu.edu.cn (Y.C.)

* Correspondence: yuelingbai@shu.edu.cn (Y.B.), xujiaqiang@shu.edu.cn (J.X.)

† These authors contributed equally to this work.

EXPERIMENTAL DETAILS

Copper (II) nitrate heMi (pentahydrate) ($Cu(NO_3)_2 \cdot 2.5H_2O$, 98.0%) and 1,3,5-Tri(4-carboxyphenyl) benzene (H_3BTB, 98.0%) were obtained from Alfa Aesar. Benzene (99.0%), toluene (99.5 %), xylene (99.0 %) and N, N-dimethylformamide (DMF, 99.0%) were purchased from Shanghai Chemical Reagent Co., Ltd. Methanol and sodium acetate were got from Sinopharm Co., Ltd.

Synthesis of MOF-14 with microporous structure

Synthesis of MOF-14 was performed following the reported procedures [1] with a few modifications. First, 43.8 mg H_3BTB (0.1 mmol) and 46.4 mg $Cu(NO_3)_2 \cdot 2.5H_2O$ (0.2 mmol) were each dissolved into 25 mL mixture of ethanol and deionized water with volume ratio 4:1. Then, the acid solution was added to the copper salt solution under stirring over 10 min at ambient temperature. After stirring for 24 h, the precipitate was separated and washed with 25 mL ethanol and deionized water several times. Finally, the product was dried by Critical Point Dryers.

Characterization

Field emission scanning electron microscope (FE-SEM, JSM-6700F) was employed to observe the MOFs' morphologies and micro-structures. Powder X-ray diffraction patterns of samples were recorded at 0.02°/s and operated at 40 kV and 15 mA current with Cu $K\alpha_1$ radiation (λ=0.15406 nm) diffractometer (XRD, Dmax 2500V). The obtained N_2 adsorption-desorption isotherms were evaluated by a Micromeritics ASAP 2020 system.

Sauerbrey equation.

$$\Delta f = \frac{2f_0^2}{A\sqrt{\mu\rho}}\Delta m = -c\Delta m$$

Where f_0 (Hz) is the fundamental resonant frequency which depends on the nature of QCM chip, A (cm^2) is the area of the silver plates coated on quartz crystal. μ (dimensionless) and ϱ (g/cm^3) is the shear modulus and density of quartz crystal respectively. Meanwhile, c (Hz/g) is a related constant. The frequency shift ($\Delta f/Hz$) is proportional to the mass change of adsorption ($\Delta m/g$) on the electrode surface of the QCM.
